# Decoding the interstitial/vacancy nature of dislocation loops with their morphological fingerprints in face-centered cubic structure

**DOI:** 10.1126/sciadv.adq4070

**Published:** 2025-04-11

**Authors:** Kan Ma, Long Guo, Antoine Dartois, Estelle Meslin, Colin Ophus, Brigitte Décamps, Anna Fraczkiewicz, Alexander J. Knowles, Lumin Wang, Olivier Tissot, Frédéric Prima, Fei Gao, Huiqiu Deng, Marie Loyer-Prost

**Affiliations:** ^1^ Department of Mechanical Engineering, City University of Hong Kong, Hong Kong, China.; ^2^ Université Paris-Saclay, CEA, Service de recherche en Corrosion et Comportement des Matériaux, SRMP, F-91191 Gif-sur-Yvette, France.; ^3^ School of Metallurgy and Materials, University of Birmingham, Birmingham B15 2TT, UK.; ^4^ School of Physics and Electronics, Hunan University, Changsha 410082, China.; ^5^ National Center for Electron Microscopy, Molecular Foundry, Lawrence Berkeley National Laboratory, Berkeley, CA 94720, USA.; ^6^ Precourt Institute for Energy, Stanford University, Stanford, CA 94305, USA.; ^7^ Université Paris-Saclay, CNRS/IN2P3, Laboratoire de Physique des 2 Infinis Irène Joliot-Curie (IJCLab), Orsay, France.; ^8^ Ecole Nationale Supérieure des Mines de Saint Etienne, Centre Sciences des Matériaux et des Structures (SMS), Saint-Étienne, France.; ^9^ Department of Nuclear Engineering and Radiological Science, University of Michigan, Ann Arbor, MI 48109, USA.; ^10^ Department of Materials Science and Engineering, University of Michigan, Ann Arbor, MI 48109, USA.; ^11^ Université Paris-Saclay, CEA, Service de Recherches en Matériaux et procédés Avancés, 91191 Gif-sur-Yvette, France.; ^12^ PSL Research University, Chimie ParisTech-CNRS, Institut de Recherche de Chimie Paris, Paris, France.

## Abstract

Dislocation loops are critical defects inducing detrimental effects like embrittlement and swelling in materials under irradiation. Distinguishing their nature (interstitial- or vacancy-type) is a long-standing challenge with great implications for understanding radiation damage. Here, we demonstrate that the morphology of radiation-induced Frank loops can unveil their nature in face-centered cubic (fcc) structure: Circular loops are interstitial-type in all fcc materials, while segmented loops are vacancy-type in high stacking fault energy (SFE) alloys but varied-type in low SFE and high-entropy alloys. The polygonal shape is attributed to the dissociation of an *a*_0_/3<111> dislocation into an *a*_0_/6<112> Shockley partial and an *a*_0_/6<110> stair-rod dislocation. The dissociation of vacancy loops is energetically favorable, whereas interstitial loops require external stimuli to promote dislocation propagation. This “morphology-nature” correlation not only highlights the asymmetry of vacancy/interstitial loops but also offers an efficient way to distinguish loop nature for a wide range of materials.

## Teaser

Loop shape can reveal its underlying nature in materials, effectively offering insight into radiation damage.

## INTRODUCTION

Radiation damage in structural materials inevitably leads to substantial deterioration in both the structural integrity and material performance, including macroscopic void swelling, radiation-induced hardening, and embrittlement (*1*–*3*). These detrimental effects for structural materials are closely related to the formation of dislocation loops of opposite natures, interstitial-type (I-type) and vacancy-type (V-type), of which the formation and migration behavior remain a long-standing question (*4*, *5*).

To unambiguously determine the nature of a dislocation loop, which is a two-dimensional (2D) structural defect, transmission electron microscopy (TEM) is so far the only means currently available. The determination of the dislocation loop nature using TEM involves the dynamical theory of dislocation image contrast (*6*–*8*). Researchers developed the theory in the 1960s and have applied it to a wide range of materials (*9*–*15*). In practice, it necessitates multiple imaging to determine the Burgers vector of the loop (such as the apparent invisibility of the loop when a particular reflexion operates) to identify the loop habit plane or its inclination, and then to obtain inside-outside contrast behavior with two opposite diffraction vectors ±**g**, which is time consuming and requires substantial expertise. With the advancement of TEM, high-resolution scanning TEM (STEM) was used to distinguish the nature of dislocation loops directly based on atomic resolution micrographs, capable of observing a limited amount of dislocation loops with strict requirements on the sample preparation and thickness.

Nowadays, it is commonly accepted that dislocation loops formed under irradiation in materials are predominantly interstitial-type. However, experiments have shown that interstitial loops and vacancy loops may both form (*15*–*20*). Multilayer loop and complex dislocation loop structure can be either accumulations of loops with the same nature or mutual annihilations of loops with the opposite nature, which notably affect the long-term radiation behavior of materials (*10*, *21*–*23*). Face-centered cubic (fcc) materials, i.e., materials with an austenitic structure such as austenitic stainless steels (ASSs), have been widely used in nuclear reactors, and some high-entropy alloys (HEAs) are seen as promising candidates for nuclear applications (*3*, *24*). It is known that the agglomerations of point defects form sessile faulted loops (Frank loops with Burgers vector, **b**_F_ = *a*/3<111>). The nature of Frank loops induced by irradiation in the fcc structure is critical to understand the mechanisms of radiation effects, namely, the evolution and diffusion of point defects and the subsequent evolution of microstructure under irradiation, to design and develop radiation-resistant materials. However, screening the nature of dislocation loops and identifying loop nature during in situ irradiation experiments have become a challenging and complex task, which is of particular interest for advanced materials such as HEAs.

This work aims to reveal a correlation between the nature of dislocation loops and their morphology in fcc metallic materials: Visibly, vacancy Frank loops are polygonal (e.g., hexagonal), while interstitial Frank loops are circular. We propose such a correlation between the shape and nature of Frank loops in fcc structure and discuss the dependency of this correlation on the stacking fault energy (SFE), explaining this correlation mechanistically based on SFE and temperature conditions through systematic molecular dynamics (MD) simulations in fundamental fcc metals [aluminum (Al), nickel (Ni), and copper (Cu)]. This paper provides a way to characterize the nature of many dislocation loops using a single TEM micrograph, which greatly accelerates and advances the study of radiation effects in both fundamental and advanced materials. Furthermore, this correlation may extend beyond fcc structures, as both circular and hexagonal loop shapes were observed in hexagonal close-packed (hcp) materials (*25*–*27*), opening perspectives for studying dislocation loop nature in a wider range of materials.

## RESULTS

### Shape of vacancy-type Frank loops in irradiated Ni

**Fig. 1.**
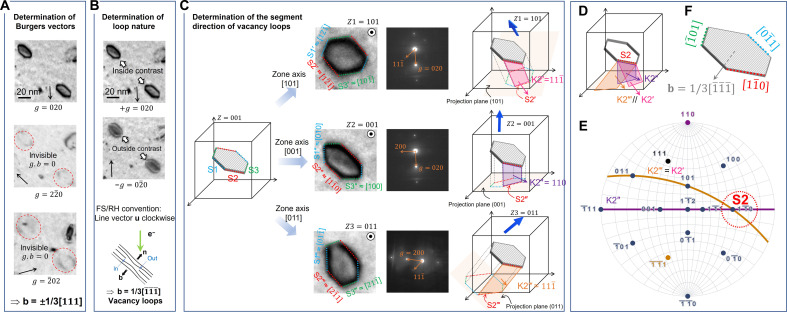
Vacancy-type Frank loop in self-ion irradiated Ni at 510°C. The inside-outside method uses bright-field TEM micrographs determining (**A**) the Burgers vector of loops and (**B**) their nature. The Burgers vector of these two loops was found to be **b** = −1/3[111] pointing downward to the loop plane normal **n** = [111], thus leading to vacancy loops. (**C**) Determination of the directions of the segments of hexagonal Frank loops. The studied dislocation loop was imaged along three zone axes **Z1** = [101], **Z2** = [001], and **Z3** = [011] under two-beam condition using **g** = {200}. Three directions of the hexagon segments are indicated as S*n* (*n* = 1, 2, 3). Their projected segments along different poles are marked as **S*n*′**, **S*n*″**, and **S*n*‴** for **Z1**, **Z2**, and **Z3**, respectively. **K*n*** = **Z*n***∧**S*n*** is the vector perpendicular to the hexagon segment and the projected segment. (**D**) The intersection of **K*n*′**, **K*n*″**, and **K*n*‴** planes is the hexagon segment with the direction determined using stereo-projection in (**E**). S2 is plotted as an example. (**F**) All segments are parallel to <110> directions.

Ni as an fcc model material was irradiated by self-ions and electrons to create dislocation loops. Figure 1A presents two typical Frank loops in self-ion irradiated Ni at 510°C and shows the determination of their nature using the inside-outside method (*9*, *21*–*23*). The three TEM micrographs in Fig. 1A show the visibility of the loops with three different diffraction vector **g**. On the basis of the invisibility criteria, these loops have the same Burgers vector colinear with the [111] direction, suggesting that their Burgers vector is **b** = ±*a*/3[111]. Thus, they are Frank loops, consistent with the fact that stacking fault contrast is shown inside the loops. In addition, the habit plane vector is **n** = [111], as Frank loops have pure edge character (*28*). The two micrographs in Fig. 1B are a ±**g** pair showing the inside-outside behavior. Diffraction vector **g** = ($0\bar{2}0$) yields an outside contrast as it is diffusing toward outside and the width of loops is larger than the one with −**g** = (020). This inside-outside behavior suggests **g**.**b** > 0, leading to **b** = *a*/3[$\bar{1}\bar{1}\bar{1}$]. Thus, the fact that the normal of loop habit plane **n** and the Burgers vector pointing to the opposite direction, i.e., **n**.**b** < 0, demonstrates the vacancy nature of these loops. These vacancy-type Frank loops have a characteristic hexagonal shape. To determine the segment directions, we projected the loops along the three zone axes as depicted in Fig. 1C. Taking the segment S2 of one loop from Fig. 1A as a representative example, we use coordinate systems based on the diffraction pattern to index the direction of the projected segment, yielding **S2′** = [$1\bar{2}\bar{1}$] (along **Z1** = [101]), **S2″** = [$1\bar{1}0$] (along **Z2** = [001]), and **S2‴** = [$2\bar{1}1$] (along **Z3** = [011]), respectively. Subsequently, for each projection, we define a vertical plane such as **K2′** by taking the vector product of **S2′** and **Z1**, resulting in **K2′** = [$11\bar{1}$]. Similar calculations yield **K2″** = [110] and **K2‴** = [$11\bar{1}$]. As illustrated in Fig. 1D, the intersection of these three vertical planes corresponds to the segment **S2**. Using the stereo-projection in Fig. 1E, the intersection of the three vertical planes is [$1\bar{1}0$]. The same analysis reveals that all segments of the hexagonal loops are parallel to <110> directions (Fig. 1F).

### Shape of interstitial-type Frank loops in Ni and fcc HEA

**Fig. 2.**
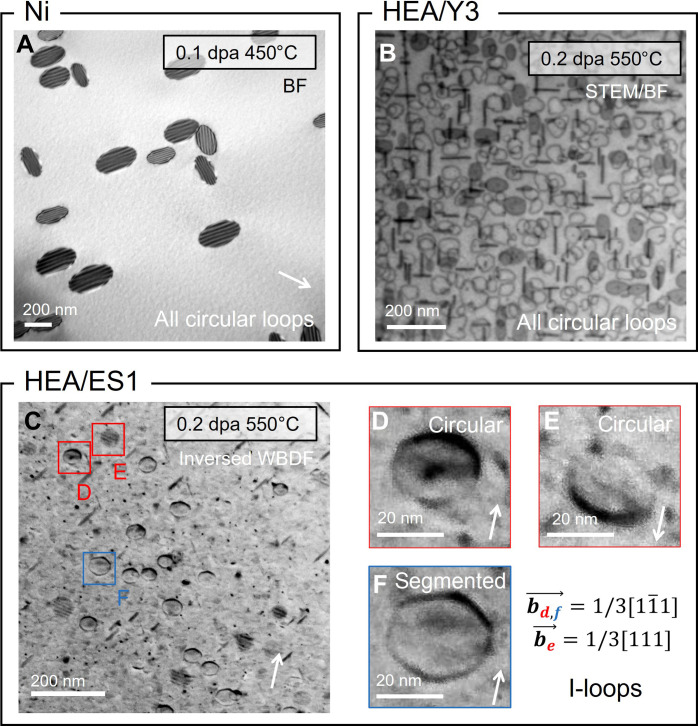
Interstitial-type Frank loop in irradiated Ni and fcc HEAs. (**A**) Ni irradiated at 450°C using a 1-MeV electron beam. HEA (**B**) Y3 (Cr_15_Fe_46_Mn_17_Ni_22_) and (**C**) ES1 (Cr_16_Fe_37_Mn_13_Ni_34_) at 550°C irradiated at 550°C using 2-MeV Fe^+^ ions. (**D** to **F**) Interstitial-type Frank loops (I-loops) with different shapes in ES1. Red rectangles in (C) mark out segmented loops in the area. Micrographs (A and C) were taken using **g** = {020} close to the zone axis [101] and (B) along the zone axis [001].

Figure 2 presents interstitial-type Frank loops in Ni and fcc HEAs. In electron-irradiated Ni, interstitial-type Frank loops were observed (see fig. S1 for determination), and they exhibit a circular or ellipsoidal shape (Fig. 2A). The ellipsoidal shape is due to the image plane not being parallel to the habit plane. Two cobalt-free fcc HEAs, Y3 (Cr_15_Fe_46_Mn_17_Ni_22_) and ES1 (Cr_16_Fe_37_Mn_13_Ni_34_), were irradiated using iron ions at 550°C to 0.2 displacement per atom (dpa). In both HEAs, only interstitial-type loops were identified (see fig. S2). Frank loops are all circular in Y3 (Fig. 2B) and mostly circular in ES1 (Fig. 2, D and E), while some manifest segmentation (Fig. 2F).

### Frank loop morphology-nature correlation in fcc materials

**Fig. 3.**
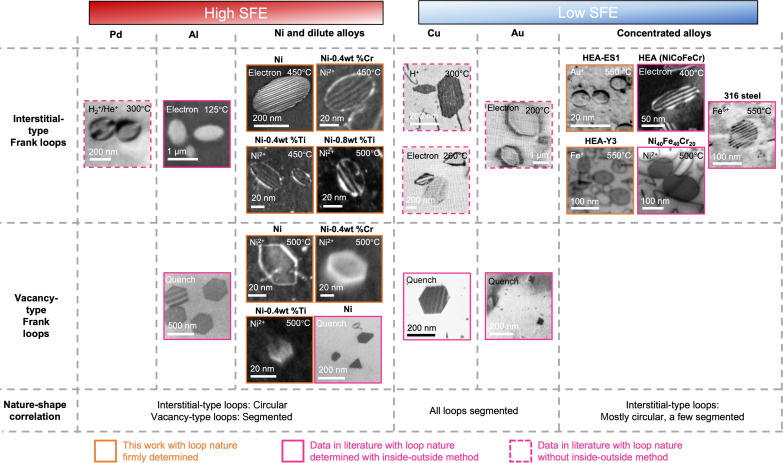
Correlation of the nature and the shape of radiation-induced Frank loops in fcc materials. TEM micrographs showing the shape of Frank loops in irradiated fcc HEA Y3 and ES1 and Ni with its dilute alloys (this work), quenched Ni (*29*), as well as Pd (*78*), Al (*11*, *31*), equi-atomic NiCoFeCr HEA (*32*), concentrated solid-solution alloys Ni_40_Fe_40_Cr_20_ (*33*), austenitic steels 316ASS (*34*), Cu (*12*, *13*, *35*), and Au (*35*, *36*). Images adapted from the cited publications. The conditions of loop creation (ion/temperature or quenching) are shown in each image. Inset figures of Pd, Al, Cu, Al, Ni, 316ASS, NiCoFeCr HEA, and NiFeCr alloys reproduced from (*11*, *32*–*34*, *78*), (*12*, *13*, *31*, *36*), and (*35*) with permission from Elsevier, Taylor & Francis, and the Physical Society of Japan, respectively.

The observations in Ni and HEAs suggest a link between the shape and nature of Frank loops in fcc materials. Figure 3 summarizes the nature (interstitial-type or vacancy-type) and shape (circular or polygonal) of Frank loops in this work and from the literature (*11*–*13*, *29*–*36*). These Frank loops were created either by irradiation using ions/electrons or by quenching. Those with their nature firmly determined under the same Finish-Start/Right-Hand (FS/RH) convention are outlined with solid lines, while data with assumed nature or ambiguity are outlined with a dashed line. In palladium (Pd) (*30*), Al (*11*, *31*), and Ni and its alloys [in this work and in (*29*)], a correlation between the loop shape and nature is observed. Across compositionally complex fcc alloys including equi-atomic NiCoFeCr HEA (*32*), Ni_40_Fe_40_Cr_20_ (*33*), and ASSs (*34*, *37*), to our knowledge, only circular interstitial-type Frank loops induced by irradiation were reported before unfaulting occurs. In other pure metals such as Cu (*12*, *13*, *35*), silver (Ag) (*12*, *35*), and gold (Au) (*36*), both interstitial and vacancy loops were found to be segmented.

Briefly, a general correlation between the shape and nature of Frank loops is revealed: Vacancy-type Frank loops tend to develop a threefold symmetry, such as a hexagonal shape, with segments parallel to <110> directions, while interstitial-type Frank loops are circular or ellipsoid in most cases except Cu and Au. Some interstitial-type Frank loops in HEAs also exhibit a segmented shape. However, the data provided in irradiated Cu (*13*, *35*) and Au (*35*) remain ambiguous to confirm the interstitial nature of the segmented Frank loops. Some SFE data of the considered metals and alloys measured by TEM techniques are given in Table 1. The correlation appears independent of the dislocation loop generation conditions (irradiation or quenching), irradiation type, and composition but does show a dependence on the SFE of the material.

**Table 1.** SFE of fcc materials based on TEM weak-beam techniques.

Materials	Intrinsic SFE (mJ m^−2^)
Ag	16 (*79*, *80*)
Au	32 (*81*)
Cu	42 (*80*), 45 (*43*)
ASS	20–35 (*65*)
fcc-HEAs	27–38 (*67*)
Ni	120–130 (*82*)
Al	166 (*44*)
Pd	180 (*83*)

### Favorable shape based on the loop formation energy

**Fig. 4.**
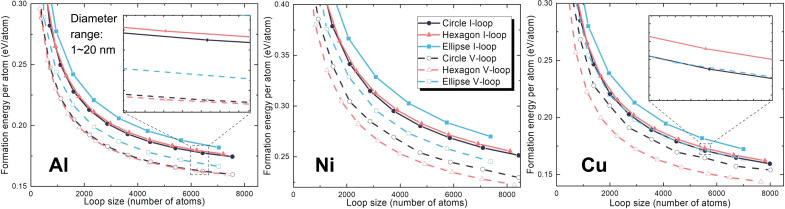
The formation energy of the interstitial-type and the vacancy-type Frank loops of various shapes in Al, Ni, and Cu. In all three metals, the hexagonal shape has the lowest formation energy for vacancy Frank loops, and the circular shape has the lowest energy for interstitial Frank loops. I-loop stands for interstitial loops, and V-loop stands for vacancy loops.

A faulted loop consists of the stacking fault and the dislocation line at the periphery. Here, the correlation is counterintuitive as, for a given loop area, the circular loop should be the lowest energy configuration, where the dislocation line tension is minimal regardless of the loop nature. To understand the mechanism behind this morphology-nature correlation, we calculated the formation energy of Frank loops in Al, Ni, and Cu using the embedded-atom model (EAM) potential by Liao *et al.* for Al (*38*) and Mishin *et al.* for Ni (*39*) and Cu (*40*) (Fig. 4). MD calculation of the SFE using these potentials yields a reasonable agreement with the density functional theory (DFT) calculations (*41*, *42*) and the experimental values (*43*–*45*) (see fig. S3). Various shapes of loops of both natures were considered (see fig. S4). The MD simulations suggest that, for vacancy loops, the hexagonal shape is energetically favorable, and, for interstitial loops, the circular shape has the lowest formation energy. The results are consistent with the experimental observations except that Frank loops in irradiated Cu and Au presumed to be interstitials were found segmented. Therefore, to validate the correlation, it is crucial to investigate (i) the origin of the hexagonal shape of vacancy loops leading to the difference between loop natures and examine (ii) the nature of Frank loops in irradiated Cu and Au and (iii) the interplay between the SFE and the shape of interstitial Frank loops.

### Hexagonal shape of vacancy Frank loops driven by dislocation dissociation

**Fig. 5.**
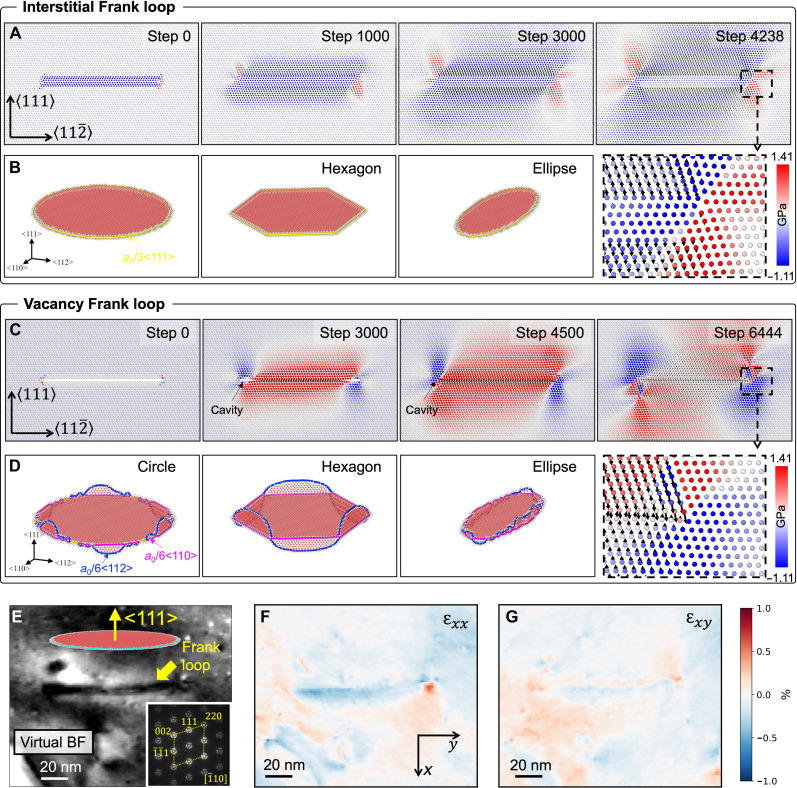
Stress distribution around Frank loops with opposite natures in Ni. Maps of hydrostatic stress along <111> direction around (**A**) interstitial Frank loop and (**C**) vacancy Frank loop during the relaxation process as a function of relaxation steps. Representative steps were selected to illustrate the strain field evolution. The diameter of simulated loops is 6 nm. The color code of atoms in (A) and (C) is white for no stress, blue for compression, and red for tension. After relaxation, the stable loop configuration is shown for (**B**) the interstitial loop and (**D**) vacancy loop. Around the vacancy loop, the high shear stress induces dislocation dissociation shown in (D). The loops’ structure was examined by dislocation extraction algorithm (DXA) analysis. Dislocations in (B) and (D) with a Burgers vector of *a*_0_/3<111>, *a*_0_/6<112>, and *a*_0_/6<110> are colored in yellow, blue, and pink, respectively. (**E**) 4D-STEM virtual bright-field (BF) image of an interstitial-type Frank loop in Ni-0.4Ti irradiated at 450°C using 5-MeV Ni^2+^ ions up to 0.7 dpa, with corresponding hydrostatic strain maps of $\varepsilon_{\mathrm{xx}}$ and $\varepsilon_{\mathrm{xy}}$. Inset image in (E) is the average diffraction pattern of the 4D-STEM dataset. Red and blue in (**F**) and (**G**) represent tension and compression, respectively.

Figure 5 elucidates the nucleation process and structure of interstitial and vacancy Frank loops by MD simulation. The atoms in Fig. 5 (A and C) are color coded to represent their stress state: Green indicates atoms without stress, blue indicates atoms under compressive stress, and red indicates atoms under tensile stress. The atoms of the extra plane in an interstitial Frank loop push the surrounding atoms away by exerting a compression stress field around the loop as shown in Fig. 5A. 4D-STEM was used in an irradiated Ni–0.4 wt % Ti (Fig. 5E) to investigate the strain distribution around an edge-on interstitial-type Frank loop. Consistent with the MD simulation, a low compression field is observed inside the faulted plane, while, at the edge, a concentrated hydrostatic strain exists with a slight shear strain (Fig. 5, F and G). As for vacancy loops, their formation is initiated by the collapse of surrounding atoms. This process begins internally and progresses toward the loop’s edge, forming an unstable “cavity” characterized by high local stress depicted in Fig. 5C. Unlike the case of an interstitial loop, the collapse of this cavity leads to high shear stress at the periphery of the vacancy Frank loop, triggering the activation of favorable slip systems and leading to the nucleation and propagation of Shockley dislocations depicted in Fig. 5D. A notable outcome of this process is the dissociation of an *a*_0_/3<111> dislocation segment into an *a*_0_/6<112> Shockley and an *a*_0_/6<110> stair-rod dislocation segment (Fig. 5D), which is not observed for interstitial loops (Fig. 5B). These dissociation phenomena have been suggested for stacking fault tetrahedra in quenched Au (*46*) and recently in irradiated austenitic steels (*47*) and Pd (*48*). In Fig. 5D, we show that the degree of dissociation varies with the loop’s geometry: Hexagonal loops exhibit the highest degree, followed by circular and elliptical ones. Furthermore, the dissociation releases the strain at the dislocation line lowering the formation energy of the loop, suggesting that configuration stability increases with the dissociation degree. This degree of dissociation can be indicative of the propagation distance of the Shockley dislocation. The motion of Shockley dislocations serves to alleviate stress, suggesting that high dissociation degrees effectively facilitate stress relief during the nucleation of vacancy loops, thereby contributing to lower formation energies.

Our investigation extends to the energetic states of hexagon-shaped vacancy loops at various rotation angles on the {111} planes. By rotating the segment away from the position parallel to the <110> directions, the dissociation degree in these hexagonal loops diminishes (see fig. S6A). Notably, loops with <110> segments exhibit maximal dissociation, while loops with <112> segments show minimal dissociation. This variation in dissociation degrees implies that hexagonal loops are energetically favorable, as further evidenced by the residual stress state (RSS) release and formation energy (fig. S6B). It reveals an inverse relationship between the RSS release value and the formation energy of vacancy Frank loops. Consequently, loops with <110> segments are the most energetically favorable, attributed to their highest RSS release values. This finding aligns with the experimental observations of the hexagonal vacancy Frank loops (Fig. 1) in our irradiated Ni samples and in quenched Al with triangle vacancy Frank loops (*49*).

In contrast, circular or elliptical vacancy loops that can be conceptualized as polygons with an infinite number of sides should have much higher formation energy, as the proportion of <110> segments is lower than <110>-segment hexagonal loops. Under identical loop sizes, circular or elliptical loops should exhibit lower stress release values than their hexagonal counterparts. This leads to the conclusion that the hexagonal shape represents the most energetically favorable configuration for vacancy Frank loops, which explains the constant experimental observation of threefold polygonal shape of vacancy loops.

### Influence of the SFE on the morphology-nature correlation

MD simulations in Fig. 4 indicate a consistent pattern between experiment and modeling: The circular-shaped interstitial loops remain the most energetically favorable configuration, regardless of the potential used (fig. S3). A key distinction between interstitial loops and vacancy loops is the distribution of stress as illustrated in Fig. 5. Around interstitial loops, stress concentrates along the faulted plane instead of at the loop’s edge, not triggering the dissociation of the 1/3<111> dislocation, as depicted in Fig. 5B. Consequently, the shape of the interstitial loop, whether circular or hexagonal, does not effectively trigger the dislocation dissociation. However, among different shapes, the circular loop exhibits the minimum dislocation line that minimizes the elastic interaction with the matrix. As a result, the circular interstitial loop, with its minimal interaction area, is less prone to dissociation. Thus, it can be concluded that the circular-shaped interstitial loop, backed by its low formation energy and minimal interaction with surrounding atoms, represents the most stable structure.

Nevertheless, Fig. 3 reveals that interstitial Frank loops may not exhibit a consistently circular morphology for metals with low SFE such as Cu and Au (*12*, *13*, *35*) and/or concentrated alloys (ES1 in Fig. 2C). On one hand, the loop nature in irradiated Cu and Au can be debatable and warrants further study. There has been a long-standing consensus that, in fcc metals and alloys, dislocation loops growing under irradiation predominantly exhibit an interstitial nature, as per rate theory with dislocation bias model (*50*–*53*). However, these studies on ion-irradiated Ni and its alloys have observed the growth of vacancy loops, challenging this prevailing understanding. A recent work revised the historically assumed mechanism of interstitial defect formation in fcc metals (*54*). The authors proposed an alternative perspective, suggesting that interstitial loops in fcc structures form through the transformation of 3D compact clusters with a structure resembling A15 Frank-Kasper nanophases rather than directly originating from cascades. This new dislocation loop formation mechanism contingent upon the SFE successfully explained the irradiated microstructure with multiple damage zones in Ni (*55*). This mechanism is unfavorable to the growth of interstitial Frank loops in Cu, which is in line with the observation that, in Cu, loops created under He^+^ or Cu^+^ ion irradiation were mostly prismatic (*56*). Therefore, because of the ambiguity of the loop nature in Cu and Au, additional experimental evidence is essential to ascertain the validity of the correlation in low SFE metals.

**Fig. 6.**
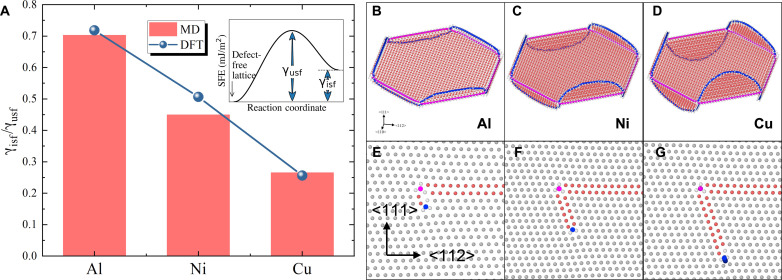
Influence of the SFE on the dislocation dissociation. (**A**) Ratio of the intrinsic SFE to the unstable SFE for Al, Ni, and Cu. Diagram showing the dissociation of dislocation segments and the structure of a vacancy Frank loop in (**B** and **E**) Al, (**C** and **F**) Ni, and (**D** and **G**) Cu, respectively. In (E) and (F), dislocations with a Burgers vector of *a*_0_/6<112> and *a*_0_/6<110> are colored in blue and pink, respectively.

On the other hand, the morphology-nature correlation may be affected by the SFE, which will affect the dislocation dissociation. The intrinsic SFE ($\gamma_{\text{isf}}$) is known as the energy difference between a perfect lattice and one with an intrinsic stacking fault. However, during the creation of a stacking fault by atomic slip, the lattice will undergo a high energy status, as shown in the schematic inset figure in Fig. 6A and fig. S3. This creates an energy barrier to nucleate a partial dislocation (*57*), defined as the unstable SFE ($\gamma_{\text{usf}}$). The $\gamma_{\text{isf}}$ and $\gamma_{\text{usf}}$ for Al, Ni, and Cu by MD calculations are respectively $\gamma_{\text{isf}}$ = 145/136/45 mJ m^−2^ and $\gamma_{\text{usf}}$ = 207/303/167 mJ m^−2^. Although $\gamma_{\text{usf}}$ and $∆\gamma \equiv \gamma_{\text{usf}}-\gamma_{\text{isf}}$ are sometimes described as energy barriers for activating stacking faults (*58*, *59*), we select the ratio between the intrinsic SFE and unstable SFE value, $\gamma_{\text{isf}}/\gamma_{\text{usf}}$, as a characteristic measure of materials, as it has been considered as a robust and universal scaling parameter rather than the absolute values to describe the dislocation behavior and deformation mechanisms (*58*–*61*). For example, the absolute values of $\gamma_{\text{usf}}$ and ∆γ varied substantially depending on the potential used, yet the ratio remained consistent and reliably predicted the dislocation behavior in fcc metals (*60*). In our work, the value of $\gamma_{\text{isf}}/\gamma_{\text{usf}}$ decreases in order of Al, Ni, and Cu (Fig. 6A). The effect of SFE on the vacancy loop dissociation is revealed by the increasing degree of dislocation dissociation in the order of Al, Ni, and Cu shown in Fig. 6 (B to D). A high $\gamma_{\text{isf}}/\gamma_{\text{usf}}$ ratio suggests that atoms remain in a relatively high energy state post-slip, which hinders the energy transfer to subsequent sets of slip atoms, such as the results shown in vacancy loops in Fig. 6 (E to G). This leads to a reduced slip distance for Shockley dislocations. Correspondingly, the RSS release values are expected to increase, while the formation energy decreases in the same sequence. This pattern is evidenced by the widening gap in formation energy between circular and hexagonal vacancy Frank loops for Al, Ni, and Cu (Fig. 4).

To further understand the dissociation, here, we define dissociation energy as the energy difference of the loop after and before the dissociation for a given dissociation distance (*a*_0_/2<112> for vacancy loops and 3*a*_0_/4<112> for interstitial loops). The lower the dissociation energy is, the more favorable the dissociation can occur. On the basis of MD calculations (see fig. S5), the dissociation energy is negative for vacancy loops but positive for interstitial loops, suggesting that segmented vacancy loops are energetically favorable. However, the positive dissociation energy for interstitial loops implies that the external force is needed to activate the dissociation. The observations of hexagonal interstitial loops in Cu, Ag, and Au were made at temperatures higher than 125°C, while our formation energy calculations were performed at 0 K in Fig. 4. Our MD simulations at finite temperatures demonstrate that the dissociation of interstitial Frank loops in Cu occurs within a temperature range of 27° to 300°C (see movies S1 to S3), which could lead to a hexagonal shape. On the contrary, no dissociation is observed in Al or Ni, which is consistent with the circular shape of interstitial loops in both metals. This suggests that temperature or stress acts as an external driving force inducing dislocation dissociation in interstitial loops. Notably, this dissociation involves the nucleation and propagation of Shockley partials (*a*_0_/3<111> → *a*_0_/6<112> + *a*_0_/6<110>). A low value of $\gamma_{\text{isf}}/\gamma_{\text{usf}}$ indicates that the deformation is dominated by the fast-moving Shockley dislocation (*60*). Thus, the movement of Shockley and the dislocation dissociation is in favor when the ratio is small. This is in line with the fact that dissociation energy for vacancy and interstitial loops decreases when $\gamma_{\text{isf}}/\gamma_{\text{usf}}$ decreases (fig. S5) in the order of Al, Ni, and Cu. Consequently, the decreasing values of $\gamma_{\text{isf}}/\gamma_{\text{usf}}$ in Al, Ni, and Cu indicate an increasing degree of dissociation and increasing energetic stability for hexagon-shaped vacancy Frank loops, as demonstrated by experiments and simulations. MD simulations show that increasing the temperature of Al and Ni up to 400° and 600°C, respectively (both above 0.5*T*_m_, with *T*_m_ the melting point of the metal), leads to the dissociation of the interstitial loops (fig. S7). At high temperatures, the enhanced diffusivity of interstitials and the lattice thermal vibration could favor the atomic rearrangement within the loop plane and around dislocation, facilitating the dissociation and loop shape transformation. Although dissociation occurs in Al and Ni at *T* > 0.5*T*_m_, it may not be of practical interest. Al and its alloys are limited at temperatures <300°C due to their low melting point. Increasing the irradiation temperature of Ni over 600°C will enhance point defect diffusion and reduce loop formation. The present temperatures reflect typical conditions of radiation damage research, corresponding to cladding material temperature in end-applications, e.g., pressurized water reactors and some Generation IV (Gen IV) reactors.

Furthermore, note that the correlation between loop shape and nature is revealed in a “static” manner. Kinetic phenomena such as unfaulting or climbing may also affect the loop shape. Some partially unfaulted interstitial Frank loops were observed to be segmented (*37*). The segmented Frank loops that were undergoing an unfaulting process by Shockley partial and zigzagged segments on the edge were found to align along <112> directions. The unfaulting process may affect the shape of the original circular loops.

For dislocation climb, in situ irradiation at different doses has shown that the morphology of Frank loops remains the same during their growth (*23*). In contrast, the loop shape could change during shrinkage by annealing (*62*, *63*). In supersaturation conditions (irradiation and quenching), the vacancy concentration in the matrix is orders of magnitudes higher than in the vicinity of the dislocation loop, leading to an osmotic force, i.e., climb force, of several orders of magnitude higher than the internal mechanical force. The dependency of climb rate on climb force along the dislocation loop is negligible, as the supersaturation contribution is dominant over climb velocity. Therefore, loops are assumed to retain their form during the climb (growth). However, during annealing, vacancy loops shrink by the emission of vacancies from the loop to the bulk (*64*). The emission is due to climb components of the internal elastic forces within the loop (*63*). This internal climb force was found to be a function of the angular position around the loop center; thus, it is nonhomogeneous along the dislocation line and has a local maximum at the sharp corners of loops. Thus, a higher climb rate due to a higher climb force at the corner will lead to the rounding of dislocation loops and alter the shape from hexagonal to circular, as shown in Al by experiment (fig. S8A) and simulation. No observation of the shrinking of Frank loops in Ni is reported. We performed in situ annealing at 510°C on an ion-irradiated Ni, where polygonal vacancy Frank loops are detected in fig. S8B. While the segmented loops in Al became completely circular within several minutes at 140°C, the loops in Ni maintained their segmented shape after an hour of annealing at 510°C. It suggests an interplay between the nonconservative dislocation movement (e.g., climbing) and the aforementioned dislocation dissociation on the shape of Frank loops. The shape change process (from hexagon to circle) occurs faster at a lower temperature in Al than in Ni. This agrees with the dislocation dissociation mechanism, as the dissociation is favored at higher temperatures and in Ni than in Al. Last, note that, although annealing may affect the loop shape, post-annealing radiation–induced dislocation loops at the irradiation temperature will not change the loop shape, e.g., the case in Ni in fig. S7B. Therefore, along the irradiation experiments that usually end with a short cooling period, the radiation-induced dislocation loops will retain their shape. It renders this loop nature-morphology correlation of great interest to the radiation damage community.

### Loop morphology-nature correlation in HEAs

The formation energy calculations by MD, shown in fig. S9, suggest that interstitial-type Frank loops in the ES1 HEA should be circular. However, there are still a few interstitial loops exhibiting a segmented shape. The stable/unstable SFE of HEAs and medium entropy alloys (MEAs) is highly dependent on the potential for first-principles calculations. The SFE of the two HEAs studied here is 49 mJ m^−2^ for ES1 and 42 mJ m^−2^ for Y3, deduced by MD simulations using the potential developed for these Co-free HEAs, agreeing with the SFE (10 to 40 mJ m^−2^) experimentally measured by TEM experiments (*65*–*67*). These SFE values should indicate dislocation dissociation in HEAs similar to Cu and Au. The observation in ES1 suggests that some interstitial-type Frank loops tend to transform from a circular shape to a segmented shape. However, circular interstitial-type Frank loops are still the majority not only in ES1 and Y3 but also in other compositionally complex alloys (*32*, *33*). This can be attributed to the high unstable SFE and complex energy landscape in these alloys. Although HEAs show the lowest intrinsic SFE, they also exhibit the highest unstable SFE (>400 mJ m^−2^), which offers a strong energy barrier for dislocation dissociation to occur. This can also explain why few dissociation occurs in austenitic steels, where the unstable SFE is ~300 mJ m^−2^ (*68*). Furthermore, in HEAs, the short-range ordering and radiation-induced segregation in the vicinity of loops substantially affected the local SFE, leading to a complex energy landscape in the alloys (*69*). The SFE in a CrCoNi MEA could have a substantial variation of up to 70 mJ m^−2^. This complex energy landscape should also create a high energy barrier for the dislocation dissociation to propagate. However, once the dissociation is stimulated by high temperatures at the individual part of the dislocation loop, the low stable SFE in HEAs will favor the segmentation of the loop, which results in the observations of some segmented interstitial-type loops. A final note, although beyond the scope of the present work, is that no vacancy-type dislocation loops have been reported to our knowledge. On one hand, the data on the nature of radiation-induced dislocation loops in HEAs are still scarce. Radiation-induced loops in HEAs and other fcc alloys were historically assumed to be interstitial-type. On the other hand, it was shown that the interstitial clusters in HEAs exhibited short-distance 3D gliding, unlike those in pure Ni showing long-distance 1D gliding (*70*), which could suppress the excess of vacancies and vacancy loop formation.

## DISCUSSION

**Fig. 7.**
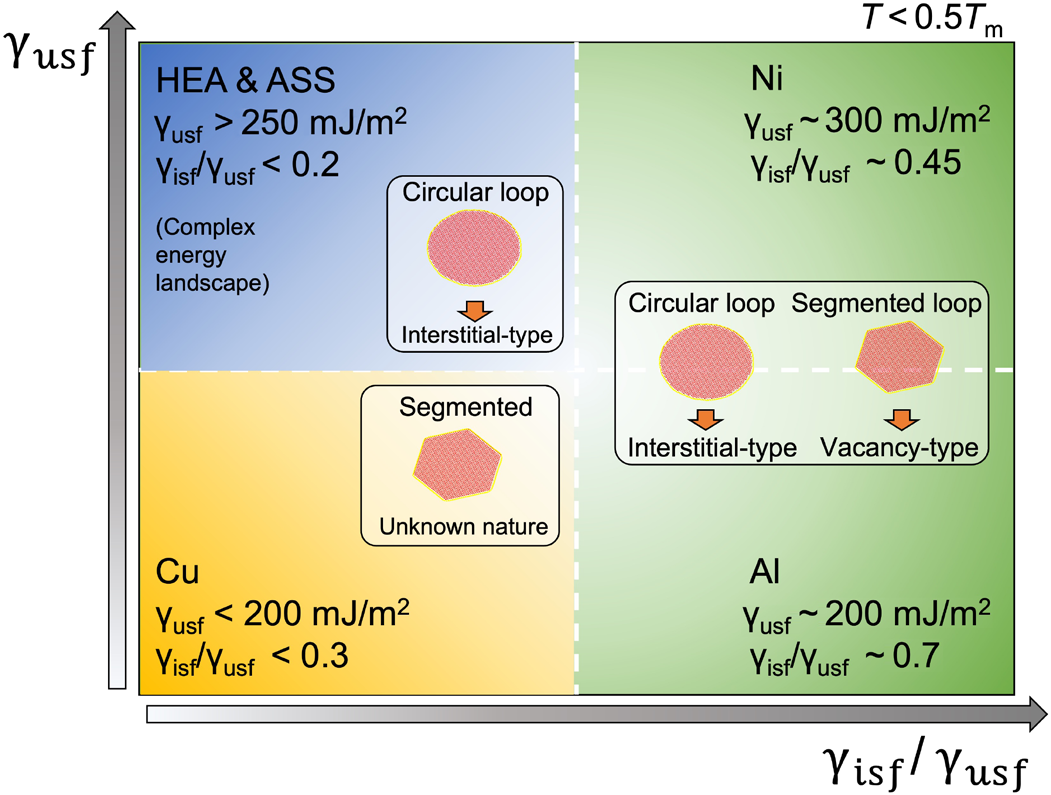
Proposed nature-morphology correlation map for radiation-induced Frank loop in fcc materials. γ_usf_, γ_isf_, and *T*_m_ are the unstable SFE, intrinsic SFE, and the melting point of the material.

We have revealed a distinct correlation between the shape and nature of Frank loops in fcc metals and alloys through a dislocation dissociation mechanism, which is dependent on the intrinsic SFE, unstable SFE, and their ratio. The correlation is validated experimentally in electron- and ion-irradiated samples. Radiation-induced Frank loops in neutron-irradiated Ni or other fcc materials lack detailed nature determination, and they were historically assumed as interstitial-type, for instance, the segmented Frank loops observed in neutron-irradiated Ni (*71*) where no loop nature characterization was performed. However, the present findings suggest that they are likely to be vacancy-type and that the nature of the dislocation loop should be carefully examined. To facilitate the determination of the Frank loop nature, this work offers a loop nature-morphology correlation map as a function of materials in Fig. 7. This correlation simplifies the characterization of loop nature in fcc materials commonly studied for fundamental radiation damage. Our MD simulations highlight the asymmetry of the strain distribution around vacancy and interstitial loops as a key factor in understanding the initiation of dislocation dissociation and its role in the morphology-nature correlation, in agreement with the 4D-STEM strain mapping. These findings not only bridge the material property, SFE, with the feature of radiation damage but also greatly streamline the determination of dislocation loop nature in fcc materials, enabling the unveiling of loop nature using a single TEM micrograph. Accurately identifying interstitial and vacancy loops is crucial as they play different roles in the modification of macroscopic properties and microstructure of structural materials under irradiation. Interstitial loops will induce radiation-induced hardening and embrittlement, while vacancy loops, as vacancy clusters, can contribute to the swelling, analogous to the role of voids and of the vacancy <c>-loops in hcp materials, whose formation is correlated to alloys’ accelerated growth (*72*). Vacancy loops may cause radiation-induced hardening and embrittlement, but this needs further investigation. Furthermore, their evolution under irradiation is different, such as their growth and unfaulting process. Thus, to get an accurate prediction of the long-term evolution of microstructure and mechanical properties, the loop nature is an essential parameter, which has been taken for granted for years due to the complexity of characterization. This finding holds a great implication for a wide range of materials to characterize their radiation damage. Future research should explore the potential generality of this correlation in different crystallographic systems and investigate loop shape under stress to extend the correlation to representative conditions of nuclear materials. It also opens opportunities for radiation damage screening in advanced materials and for the direct identification of dislocation loop nature during in situ irradiation experiments.

## MATERIALS AND METHODS

### Materials

The HEA ES1 Cr_16_Fe_37_Mn_13_Ni_34_ (atomic %) and Y3 Cr_15_Fe_46_Mn_17_Ni_22_ and high-purity Ni, Ni–0.4 wt % Ti, and Ni–0.4 wt % Cr were manufactured by cold crucible induction melting in the shape of cylinders of about 15 mm in diameter. Slides were cut and polished to 50 to 80 μm thick. Three-millimeter-diameter discs were punched out and annealed at 1000°C for 2 hours in a vacuum of 10^−7^ mbar, followed by air cooling to remove polishing-induced dislocations. Annealed discs were finally electropolished in a bath of methanol/nitric acid at −30°C to make TEM samples.

### Irradiation experiments

Three sets of irradiations were conducted within the framework of the French National network of accelerators for irradiation and analysis of molecules and materials (EMIR&A). (i) Ni (pure and alloyed) and HEA-Y3 were irradiated at the JANNUS-Orsay platform in Laboratoire de Physique des 2 infinis Irène Joliot-Curie (IJCLab) (*73*) using 2-MeV Ni-ions at 510°C up to 0.06 dpa and 2-MeV Fe-ions at 550°C up to 0.2 dpa, respectively. (ii) The HEA-ES1 and Ni alloys were irradiated at the JANNUS-Saclay platform at CEA Saclay (*74*) using 12-MeV Au-ions at 550°C up to 0.2 dpa and 5-MeV Ni-ions at 450°C up to 0.7 dpa, respectively. (iii) Ni is irradiated using 1-Mev electron irradiation at 450°C at the High-Voltage Electron Microscope (HVEM) facility [Microscope EM7 (Kratos)] at CEA Saclay. The final dose was up to 0.1 dpa.

### Microstructural characterization

A 200-kV FEI TECNAI G2 TEM with a LaB_6_ filament was used to perform TEM characterization loops in as-irradiated TEM thin foils and determine the nature of Frank. Kinetic bright-field (KBF) and weak-beam dark-field (WBDF) modes were used to optimize the defect contrast. In all TEM micrographs, *s*_g_ > 0. In situ annealing was conducted using a Gatan double-tilt heating holder. 4D-STEM experiments were carried out using the 300-kV TFS TEAM-1 microscope at the National Center for Electron Microscopy, Lawrence Berkeley National Laboratory. A 1- to 2-nm electron beam was used to capture nanobeam electron diffraction patterns at real-space positions. A bullseye-shaped C2 aperture of 10 μm was used to reshape the electron beam for precise lattice spacing measurements (*75*). Experiments were conducted at spot 6 with a 1.75-mrad semi-convergence angle and a 630-cm camera length. The monochromator lens setting was adjusted, and the probe current was maximized up to 44 pA for enhanced Bragg peak clarity. Scans with 1-nm step sizes were performed, and vacuum probe was recorded as templates for Bragg disk detection. The py4DSTEM package was used for identifying Bragg disk positions, which enabled the analysis of lattice spacing and strain analysis (*76*). The strain zero reference was obtained by averaging the lattice parameter across the area without dislocation loops.

### Calculations of formation energies

Here, we implemented MD simulations using the LAMMPS code to study the mechanism of Frank loop nature and shape. The initial interstitial and vacancy Frank dislocation loops were created by inserting or deleting atomic planes along the <111> direction. Molecular static relaxation with the conjugate gradient algorithm was performed to determine the formation energy of the Frank loop and to relax the initial configuration to an energetically favorable state. The formation energy is defined by $E_{\text{formation}}=E_{\text{loop}}-E_{\text{cohesive}}\cdot N$, where *E*_loop_ is the total energy of a loop system with *N* atoms, and *E*_cohesive_ is the cohesive energy. The formation energy of loops with various shapes (hexagonal, circular, and ellipsoidal) and various rotation angles were considered (see fig. S4).

### SFE calculations and dislocation dissociation model

SFE calculations were conducted for Al, Ni, and Cu using a simulation box with axes aligned to the [111], [−110], and [−1−12] directions. The box was divided into two parts along the [111] direction. The upper group of atoms was sheared by a distance of *a*_0_/$\sqrt{6}$[−1−12] (where *a*_0_ represents the lattice constant) in the [−1−12] direction, resulting in the formation of an intrinsic stacking fault. In addition, we provided calculations for Y3 (15Cr-46Fe-17Mn-22Ni) and ES1 (16Cr-37Fe-13Mn-34Ni) using a box dimension of 20 by 50 by 30 unit cells, which encompasses 180,000 randomly distributed atoms with the atomic potential published in (*77*). For dislocation dissociation, the simulation box consisted of a single edge dislocation with a Burgers vector of *a*_0_/3<111>, where *a*_0_ represents the lattice constant. In the box containing a single dislocation, its dimensions (*x*, *y*, and *z* in the <111>, <110>, and <112> directions, respectively) were approximately 22 by 4 by 30 nm^3^ (fig. S10). The Frank loop dislocations were created by inserting or deleting the atomic plane along the Burgers direction. After equilibration with an isothermal-isobaric (NPT) ensemble and molecular static relaxation, the dissociation-dislocation models were created. The nudged elastic band method was performed to find a minimum energy path between the two configurations. To study the dissociation as a function of temperatures, we performed MD calculation at 0/300/573/973 K on Cu, Al, and Ni (fig. S8). As the dissociation of a segment is easier than a curved line, a segmented interstitial Frank loop was chosen as the starting point.
