# Supplementary material for: Decoding the interstitial/vacancy nature of dislocation loops with their morphological fingerprints in face-centered cubic structure
Source: Sci Adv. 2025 Apr 11;11(15):eadq4070. doi: 10.1126/sciadv.adq4070 (PMC11988408; doi:10.1126/sciadv.adq4070)
Supplement: Supplementary file 1 — Figs. S1 to S10 Legends for movies S1 to S3 [file sciadv.adq4070_sm.pdf]

Science Advances
AAAS

Supplementary Materials for

Decoding the interstitial/vacancy nature of dislocation loops with their morphological fingerprints in face-centered cubic structure

Kan Ma *et al.*

Corresponding author: Kan Ma, kan.ma@cityu.edu.hk; Huiqiu Deng, hqdeng@hnu.edu.cn; Marie Loyer-Prost, marie.loyer-prost@cea.fr

Sci. Adv. **11**, eadq4070 (2025)
DOI: 10.1126/sciadv.adq4070

The PDF file includes:

Figs. S1 to S10
Legends for movies S1 to S3

Other Supplementary Material for this manuscript includes the following:

Movies S1 to S3

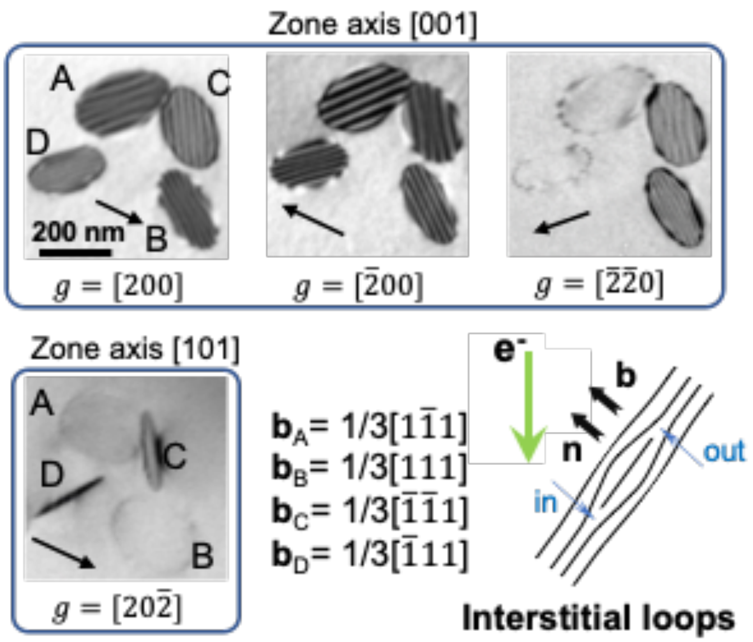

Fig. S 1 Determination of Frank loop nature in 1 MeV electron irradiated Ni at 450 °C.

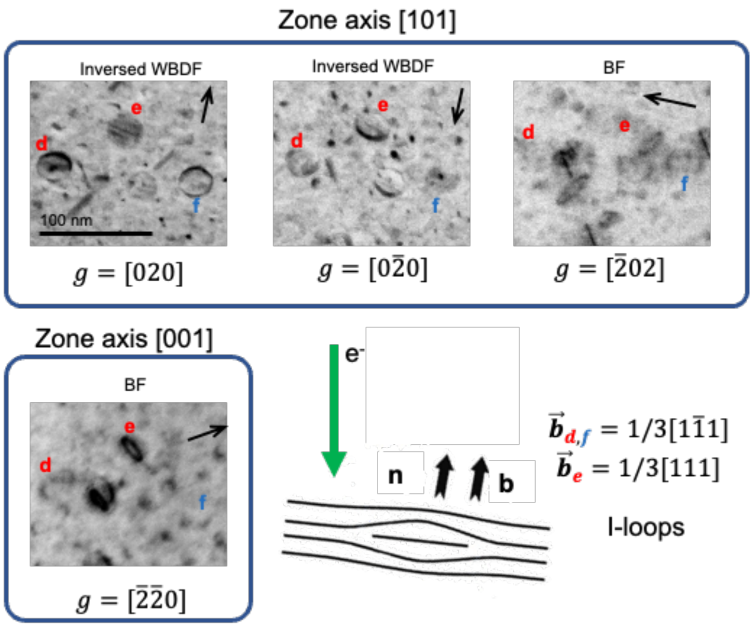

Fig. S 2 Nature of dislocation loops in ion-irradiated HEA.
Inversed weak-beam dark-field images of the Cr16Fe37Mn13Ni34 alloy irradiated at 550 °C using 2 MeV Fe^{+} ions up to 0.2 dpa.

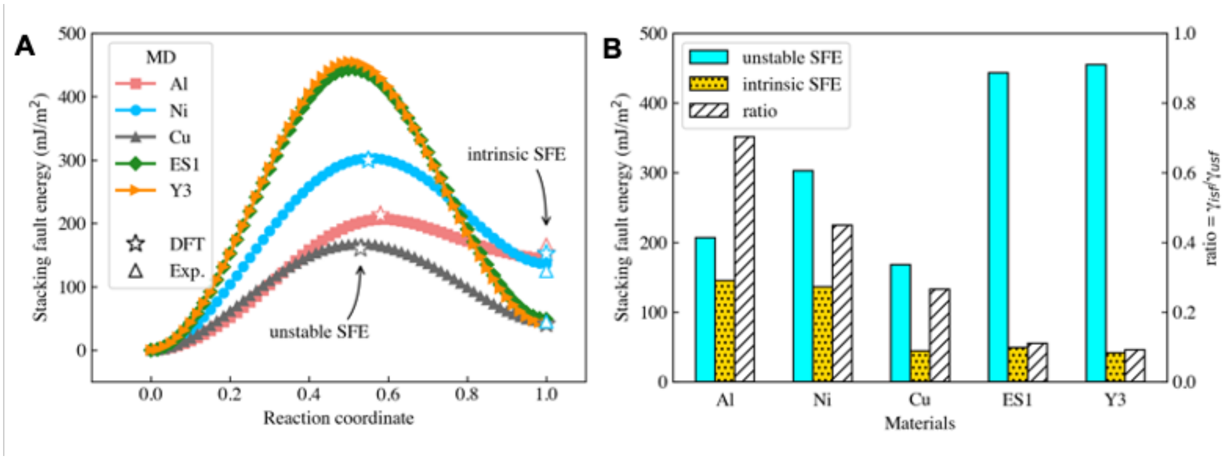

Fig. S 3 Stacking fault energies in Al, Ni, Cu and HEAs (Y3 and ES1).
(A) Stacking fault energies by the MD calculations compared with the DFT calculations (for Al *(83)*, for Ni and Cu *(78)*) and experimental data (for Al *(38)*, Ni *(39)* and Cu *(40)*). (B) Stacking-fault energies by MD of Al, Ni, Cu and HEAs. The total displacement is $a_0/6\langle 112\rangle$, where a_0 is the lattice parameter.

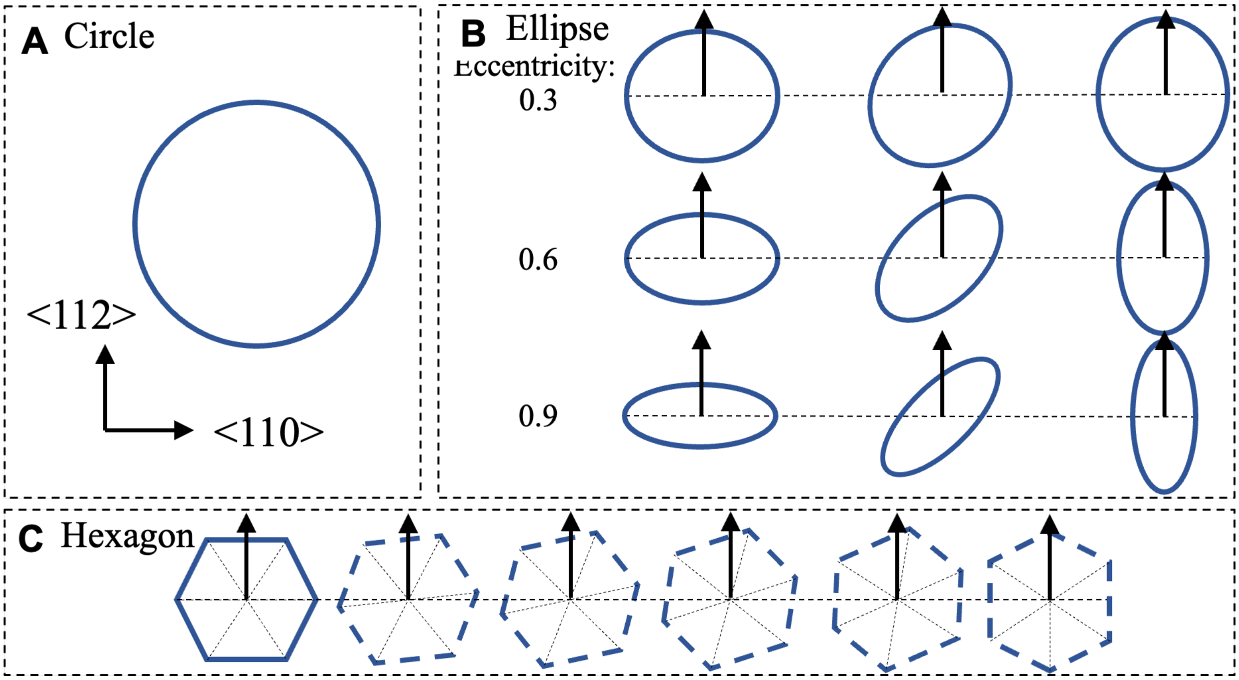

Fig. S 4 Geometry of dislocation loops for MD studies.
The initial configuration of the frank loop with varying shapes and orientations. (A) the circle shape and (B) the ellipse shape loop with different eccentricities (such as 0.3, 0.6 and 0.9) and orientations, and (C) the hexagon shape loop with different orientations. The direction of the black arrow pointing up is the <112> crystal direction.

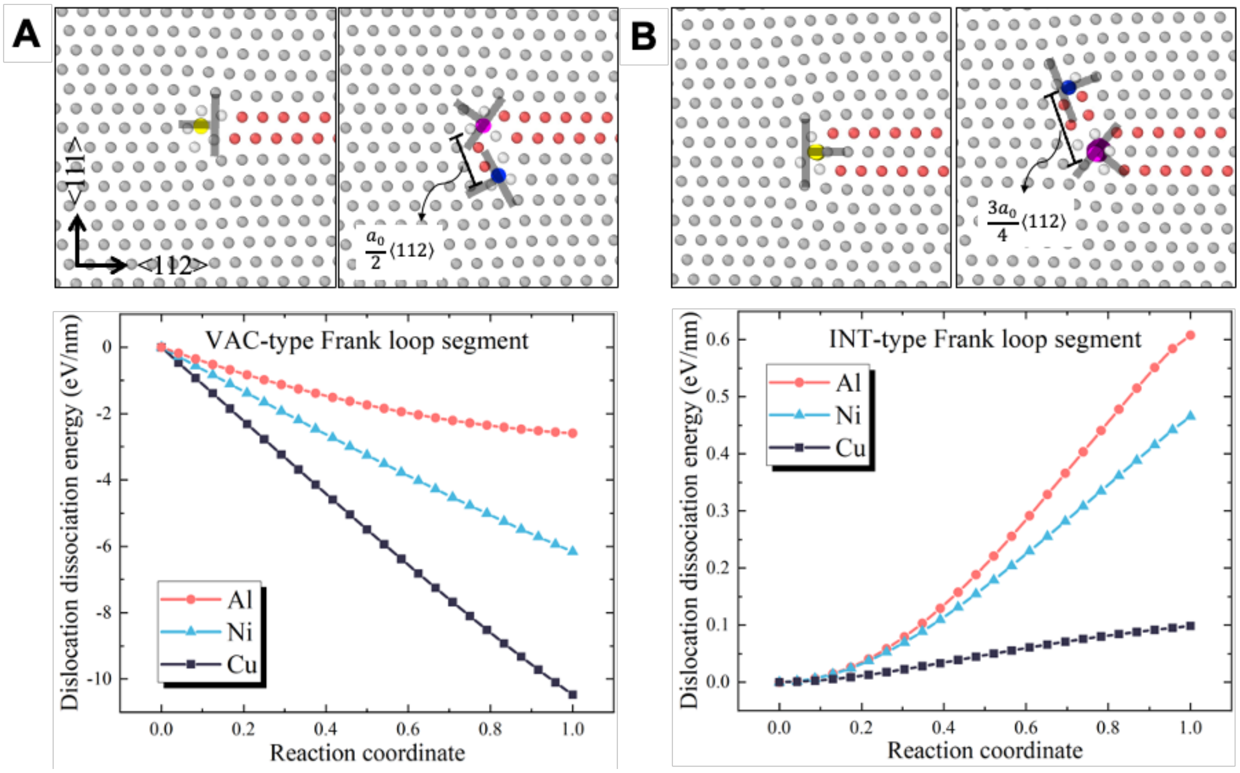

Fig. S 5 Dislocation dissociation energy in fcc in Al, Ni and Cu.

(A) case of vacancy Frank loop and (B) case of interstitial Frank loop. Dislocations with a Burger's vector of $a_0/3$<111>, $a_0/6$<112>, and $a_0/6$<110> are coloured in yellow, blue and pink respectively.

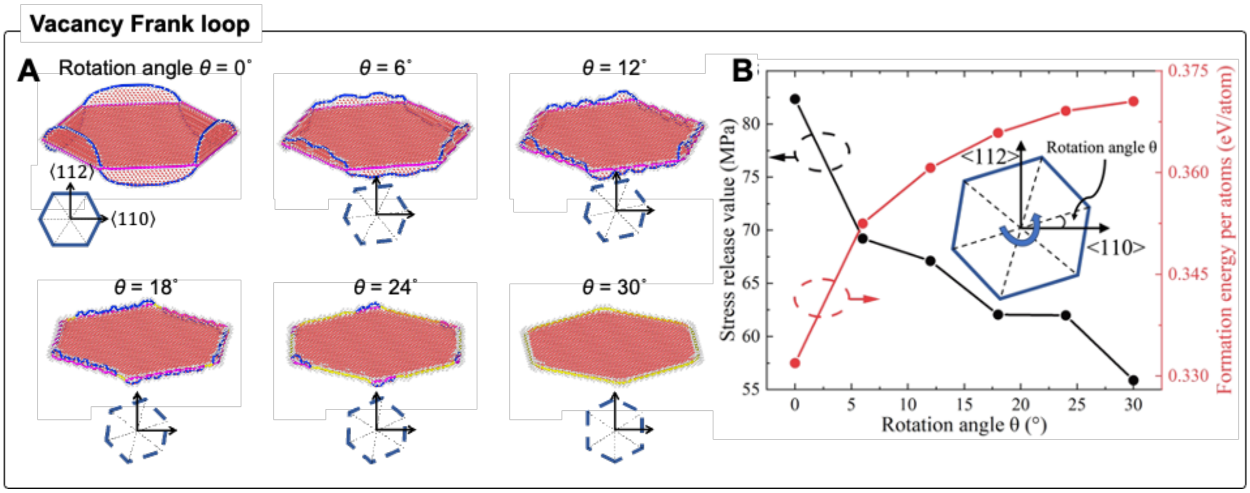

Fig. S 6 Dislocation dissociation of a hexagonal vacancy loop as a function of rotation angle. (A) Dislocation dissociation of a hexagonal vacancy loop at rotation angles θ varying from the 0° position where segments are parallel to <110> directions and to the 30° position where the segments are parallel to <112> directions. (B) The residual stress state (RSS) release and the formation energy as a function of rotation angles. The diameter of simulated loops is 6 nm. Dislocations with a Burger's vector of a0/3<111>, a0/6<112>, and a0/6<110> are coloured in yellow, blue and pink respectively.

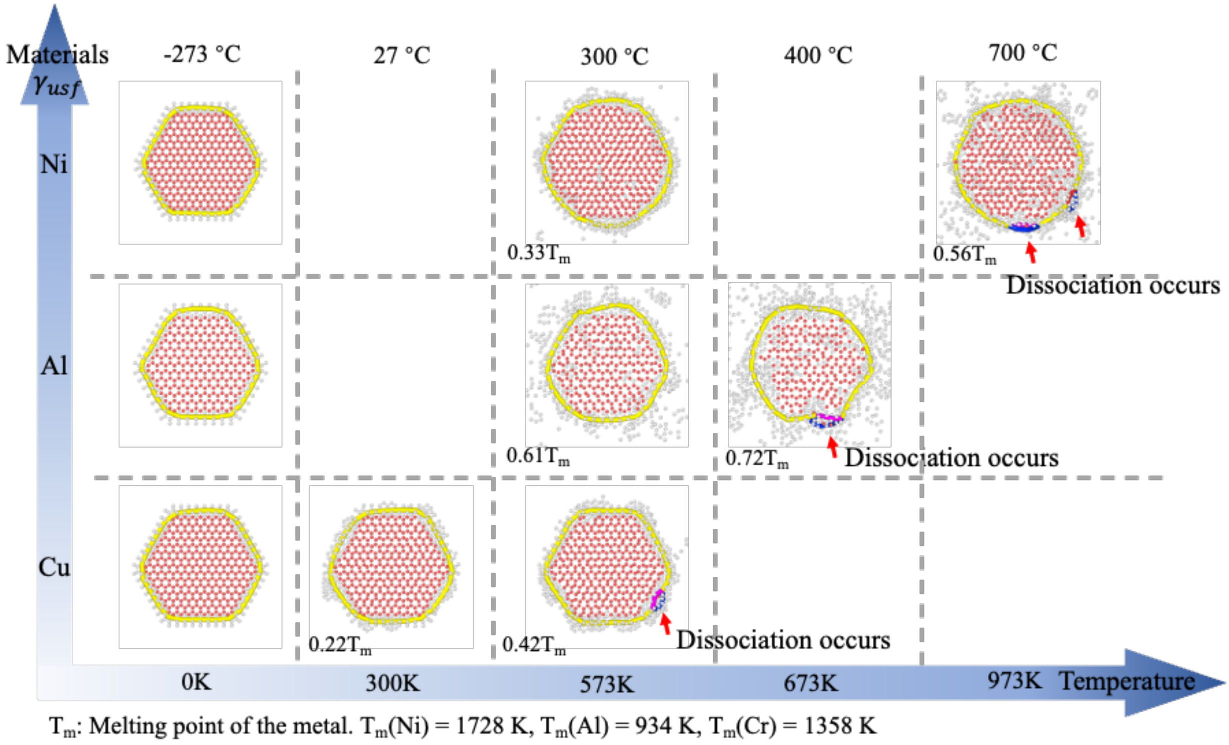

Fig. S 7 Dissociation of a hexagonal interstitial Frank loop as a function of temperature. Dislocations with a Burger's vector of a0/3<111>, a0/6<112>, and a0/6<110> are colored in turquoise, green, and red, respectively. The red toms represent the HCP structures of the fault. A segmented interstitial Frank loop was chosen as the starting point. Dislocations with a Burger's vector of a0/3<111>, a0/6<112>, and a0/6<110> are coloured in yellow, blue and pink respectively.

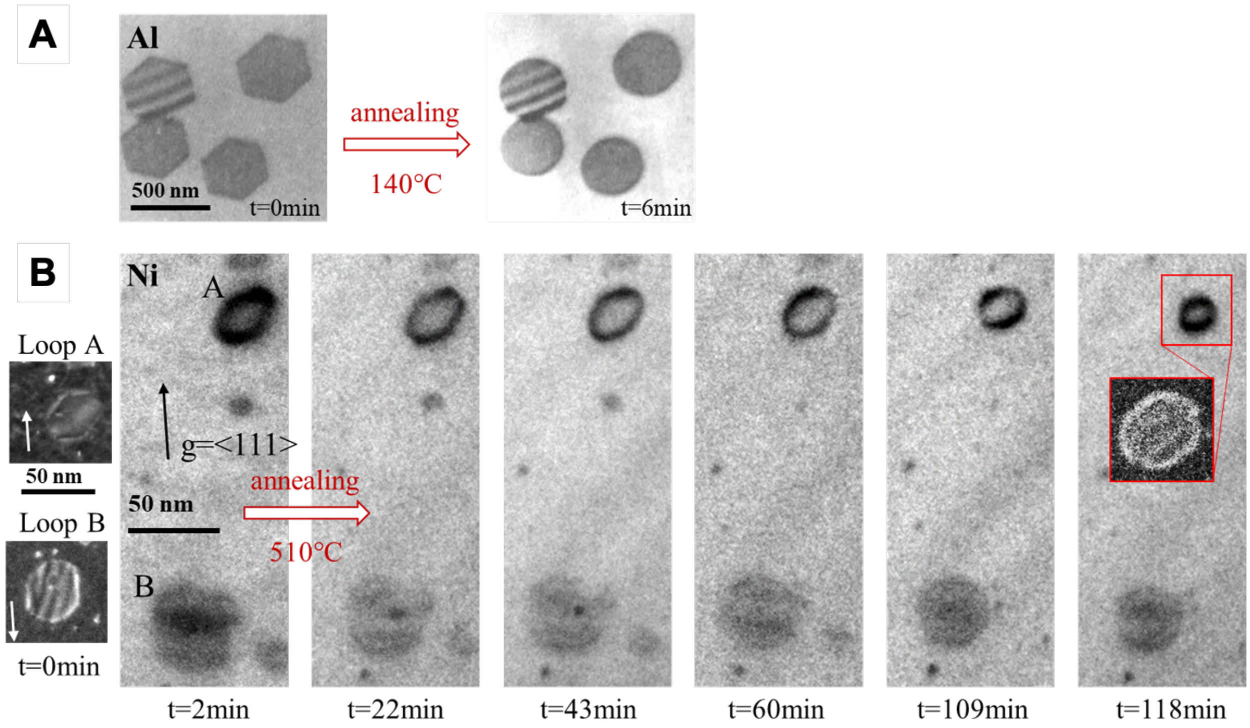

Fig. S 8 Loop shrinkage in Al and Cu.
In-situ annealing of (a) quenched aluminium *(27)* and (b) ion-irradiated Ni in TEM at 510°C in this work. In (B), bright-field image showing the evolution of two Frank loops A and B during the annealing. Weak-beam dark-field images showing the morphology of loops before and after annealing. (A) Reproduced from Philosophical Magazine, Vol 16, Issue 139, P. S. Dobson, P. J. Goodhew, R. E. Smallman, "Climb kinetics of dislocation loops in aluminium", Pages 13, 2006; with permission from Taylor & Francis under license number 5847220603975.

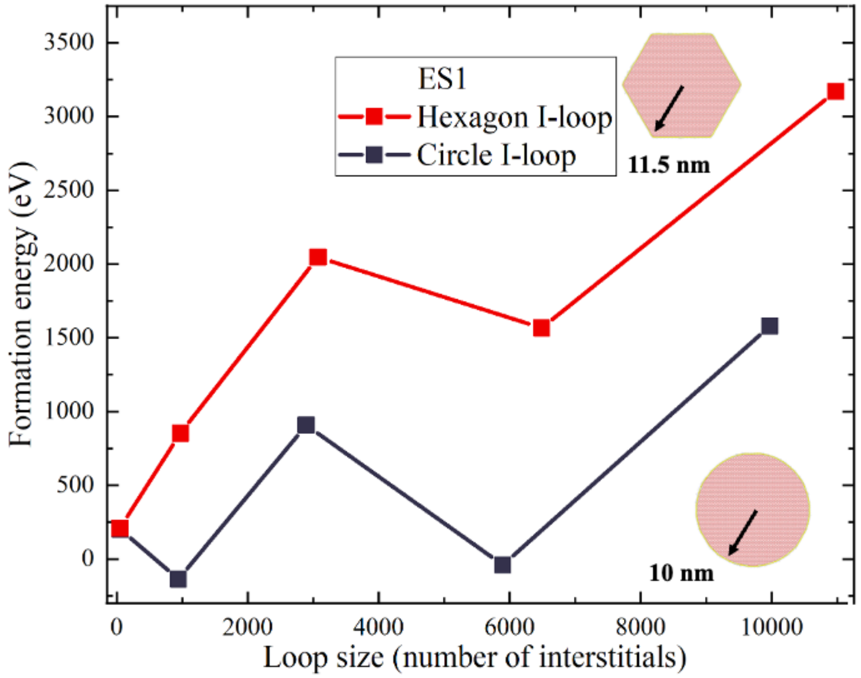

Fig. S 9 Formation energy of the interstitial Frank loops of various shapes in HEA ES1. The formation energy of the interstitial-type Frank loops of various shapes in a fcc HEA ES1 alloy showing that circular shape is the energetically stable form of interstitial-type loops in this HEA.

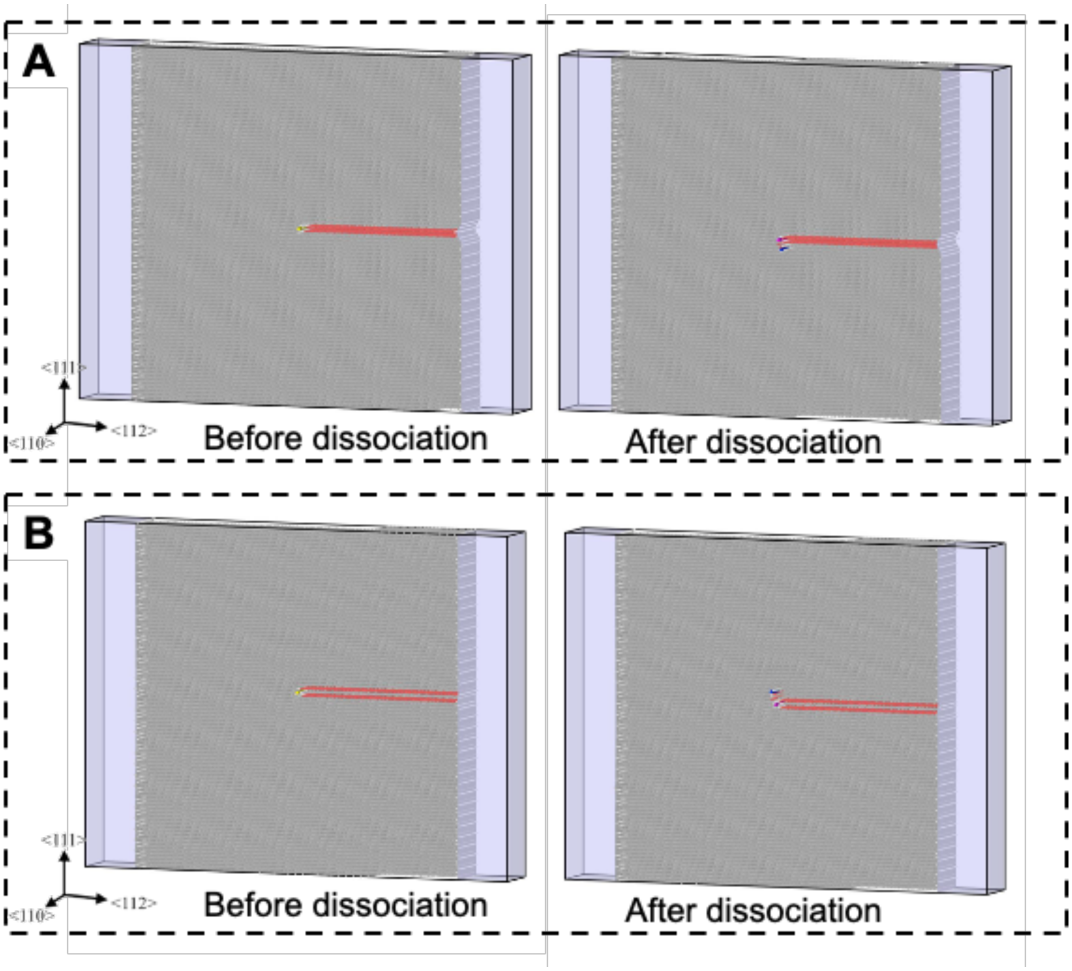

Fig. S 10 Dislocation dissociation model.
The dislocation dissociation model of a vacancy (A) and an interstitial (B) frank loop segment. Dislocations with a Burger's vector of a0/3<111>, a0/6<112>, and a0/6<110> are colored in yellow, blue and pink, respectively. The red and gray atoms represent the HCP and FCC structures, respectively.

Movie S1.
MD simulation of Frank loop dissociation at 300 °C in Cu.

Movie S2.
MD simulation of Frank loop dissociation at 300 °C in Al.

Movie S3.
MD simulation of Frank loop dissociation at 300 °C in Ni.
